# Urinary Phthalate Levels Associated with the Risk of Nonalcoholic Fatty Liver Disease in Adults: The Korean National Environmental Health Survey (KoNEHS) 2012–2014

**DOI:** 10.3390/ijerph18116035

**Published:** 2021-06-04

**Authors:** Yun-Jung Yang, Taehyen Kim, Yeon-Pyo Hong

**Affiliations:** 1 Institute of Biomedical Science, Catholic Kwandong University International St. Mary’s Hospital, Incheon 22711, Korea; yangyj@ish.ac.kr; 2 College of Medicine, Catholic Kwandong University, Gangneung-si 25601, Korea; starkim3@naver.com; 3 Department of Preventive Medicine, College of Medicine, Chung-Ang University, Seoul 06974, Korea

**Keywords:** phthalates, hepatic steatosis index, nonalcoholic fatty liver disease, Korean National Environmental Health Survey

## Abstract

The prevalence of nonalcoholic fatty liver disease (NAFLD) is increasing worldwide. Recent experimental studies suggested that phthalates might induce NAFLD. Therefore, this study aimed to investigate the relationship between phthalates metabolites and NAFLD in the human population. This cross-sectional analysis was performed using data from the Korean National Environmental Health Survey II (2012–2014) among Korean adults (*n* = 5800). NAFLD was diagnosed using the hepatic steatosis index (HSI) in the absence of other causes of chronic liver diseases. Among the participants (mean age 46 years, 47.5% male), the prevalence of NAFLD was associated with urinary levels of mono(2-ethyl-5-hydroxyhexyl) phthalate (MEHHP), mono-(2-ethyl-5-oxohexyl) phthalate, mono-(2-ethyl-5-carboxypentyl) phthalate, mono-benzyl phthalate (MBzP), and mono-*n*-butyl phthalate (MnBP) compared to the reference group. In the multivariate model, the odds ratios (ORs), 95% confidence interval (CI) for NAFLD were 1.33 (1.00–1.78) and 1.39 (1.00–1.92) in the 3rd and 4th quartile of MEHHP, respectively. Based on the study findings, high levels of urinary phthalates are associated with the prevalence of NAFLD in Korean adults. Further investigation is required to elucidate the causal relationship.

## 1. Introduction

Nonalcoholic fatty liver disease (NAFLD) is a condition whereby there is a significant accumulation of fat in the liver (>5% fat content), without excessive consumption of alcohol or other causes of liver disease [1]. The global prevalence of NAFLD is estimated to be approximately 30%, and it is likely to increase [1,2,3]. The presence of metabolic disorders, including obesity, type 2 diabetes, dyslipidemia, and hypertension, is considered to be a risk factor for NAFLD [4,5].

While a Westernized diet and sedentary lifestyle may contribute the rapid increase of the NAFLD occurrence [2,6], recent studies suggests that exposure to endocrine disrupting chemicals (EDCs) may also associated with the pathogenesis of NAFLD through insulin resistance (IR) [7,8,9]. Although IR is one of the hallmarks of NAFLD progression, EDCs can directly affect hepatic lipid homeostasis [10,11] because most of EDCs are metabolized in the liver, which is a central organ in energy metabolism.

Among the various EDCs, phthalates are the most commonly used plasticizers in industries and in daily life. Phthalates are a group of phthalic acid esters that are widely used to increase the flexibility in various consumer products including medical devices, children’s toys, personal care products, and food wrap made of polyvinyl chloride (PVC). This widespread usage of phthalate has also led to a marked increase in the exposure of the public to these agents [12]. Phthalates entering the body are rapidly hydrolyzed to the monoesters in the liver and gut [13,14,15], and most of them are excreted through the urine and feces [16,17]. Previous studies have focused on the development and reproductive toxicity after phthalate exposure [18,19]. Recent studies have suggested that phthalates can induce obesity, insulin resistance, and metabolic disorders [20,21,22,23,24,25].

The occurrence of metabolic diseases after phthalate exposure might be linked to the activation of peroxisome proliferator-activated receptors (PPARs) [25,26], which are modulated by the sterol regulatory element binding proteins (SREBPs) [27]. In experimental studies, the disruption of gene expression related to fatty acid metabolism was suggested as a plausible mechanism in the development of NAFLD after phthalate exposure [28,29,30,31,32,33,34]. However, there is a lack of clinical evidence to support the phthalates-NAFLD relationships.

Therefore, this study aimed to investigate whether urinary phthalate metabolites are associated with the development of NAFLD in the population.

## 2. Materials and Methods

### 2.1. Study Design and Participants 

This study was based on cross-sectional data obtained from the Korean National Environmental Health Survey (KoNEHS) II (2012–2014). KoNEHS is conducted every 3 years, and this is to measure the human exposure level of environmental chemicals, examine influential factors, and continuously investigate the factors of spatiotemporal distribution and changes [31]. It is a data set that includes national, multistage, stratified, and clustered probability sampling designs, so as to develop representative samples of the South Korean population.

The 6478 subjects in the KoNEHS II were aged 19 years and older (2274 men and 3704 women). They were interviewed with questionnaires on demographic characteristics, socioeconomic status, indoor/outdoor environment, lifestyle factors, and history of disease experience. Biological samples (urine and blood) were collected for clinical analysis and to measure the level of environmental chemicals. The published data of questionnaire, clinical tests, and urinary phthalate metabolite levels were used in this study.

**Figure 1:**
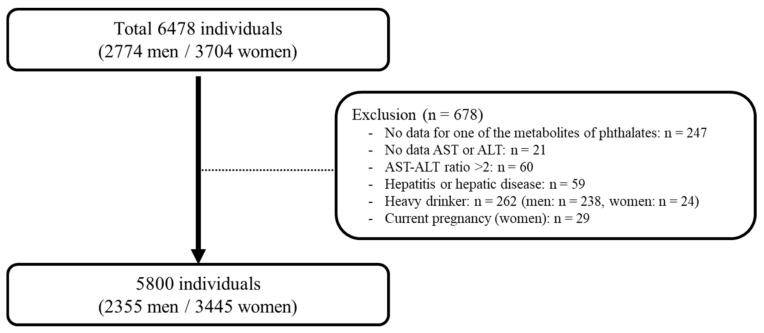
Study population in the present study obtained from the Korean National Environmental Health Survey II (2012–2014). AST: aspartate aminotransferase; ALT: alanine aminotransferase.

Based on the data, 678 participants were excluded due to a lack of data of phthalate metabolites concentrations (*n* = 247), no data of alanine aminotransferase (ALT) or aspartate aminotransferase (AST) levels (*n* = 21), significant alcohol consumption (*n* = 262, including males who consumed alcohol more than 3 times per week and 7–9 cups per time (*n* = 238) and females who consumed alcohol more than 3 times per week and 5–6 cups per time (*n* = 24)), pregnant women (*n* = 29), those with hepatitis or hepatic disease (*n* = 59), and those with an AST/ALT ratio exceeding 2 (*n* = 60). Finally, 5800 participants (2355 men and 3445 women) were included in this analysis (Figure 1).

### 2.2. Questionnaires and Anthropometric Parameters 

Data on the participants’ general characteristics, including age, sex, drinking and smoking status, physical activity, socioeconomic status, and education level, were obtained via interviews using questionnaires. These characteristics were categorized based on their responses as follows: education (less than high school, high school, and college and more), drinking and smoking (never, past, present), physical activity levels (no, moderate, yes), socioeconomic status (high, mid-high, mid-low, low), and marital status (single, married, and divorced/separated).

Participants who were diagnosed with hepatitis or fatty liver disease and who were currently undergoing treatment or taking medication were considered to have hepatic disease. Hypertension was defined as a self-reported history of hypertension or the use of antihypertensive drugs. Diabetes mellitus (DM) was defined as a self-reported history of DM or the use of antidiabetic drugs. Hyperlipidemia was defined as a self-reported history of hyperlipidemia, use of anti-hyperlipidemia drugs, triglyceride (TG) ≥240 mg/dL, or high-density lipoprotein cholesterol ≤40 mg/dL. Data on serum TG, ALT, and AST levels were obtained. Body mass index (BMI) was calculated by dividing body weight (kg) by height squared (m^2^).

### 2.3. Definition of NAFLD 

The hepatic steatosis index (HSI) was used as a screening tool for NAFLD [35,36,37]. The variables in the HSI formula were levels of ALT, AST, BMI, sex, and presence of DM. Subjects were categorized into two groups based on the HSI score: ≤36 was defined as non-NAFLD and >36 was defined as NAFLD.

$$\mathrm{HSI}=8\times \frac{\mathrm{ALT}}{\mathrm{AST}}ratio+BMI\;\left(+2,\;if\;diabetes;+2,\;if\;female\right)$$

### 2.4. Statistical Analysis 

The weighted mean or frequency and standard errors were provided. Comparisons between groups were performed using the t-test (continuous variable) or χ^2^ test (categorical variables). The concentrations of phthalate metabolites were categorized into quartiles based on the weighted sample distribution. The lowest quartile group was considered as the reference. Because the levels of phthalate metabolites were skewed to the right, log transformation was required. Multivariate logistic regression analysis was performed to predict the relationship between phthalate levels and NAFLD after considering potential demographic and clinical variables. The included demographic covariates were age, sex, smoking, drinking, exercise level, marital status, education level, and socioeconomic status. Hypertension, DM, and hyperlipidemia were included as clinical variables. In logistic regression, BMI was not used as an independent variable. The reason for this decision was that BMI is used as part of the HSI calculation formula and to avoid collinearity between BMI and HSI. However, in the HSI formula, women’s +2 is intended to compensate for women’s lower BMI compared to men. For DM, +2 was used for the same reason, so in logistic regression, sex and DM were used as independent variables. Data analyses were performed using STATA (version 15.0 StataCorp LP College Station, TX, USA). *p*-values < 0.05 were considered significant.

## 3. Results

### 3.1. General Characteristics 

The participants were categorized according to their HSI score (Table 1), with 4405 and 1395 in the non-NAFLD and NAFLD groups, respectively. The proportion of gender was not statistically different between the non-NALFD and NAFLD groups (*p* = 0.061). The mean age and BMI were significantly higher in the NAFLD group than in the non-NAFLD group (*p* = 0.005 and *p* < 0.001, respectively). The statuses of alcohol consumption were not significantly different between the two groups (*p* = 0.054); however, the status of smoking and regular exercise were significantly different (both *p* = 0.013). Socioeconomic status, education level, and marital status also showed significant differences (*p* = 0.040, *p* < 0.001, and *p* < 0.001, respectively). The proportions of participants with hypertension, DM, and hyperlipidemia were significantly higher in the NAFLD group than in the non-NAFLD group (all *p* < 0.001).

### 3.2. Urinary Levels of Phthalate Metabolites 

The distribution of urinary phthalate metabolites in the participants is shown in Table 2. Unadjusted urinary mono-(2-ethyl-5-hydroxyhexyl) phthalate (MEHHP) and mono-(2-ethyl-5-carboxypentyl) phthalate (MECPP) levels in the NAFLD group was significantly higher than those in the non-NAFLD group (both *p* < 0.001). The urinary levels of mono-(2-ethyl-5-oxohexyl) phthalate (MEOHP) was significantly higher in the NAFLD group compared to the non-NAFLD group (*p* = 0.031). Mono-*n*-butyl phthalate (MnBP), and mono-benzyl phthalate (MBzP) in urine did not differ between the groups (*p* = 1.000 and *p* = 0.085, respectively).

### 3.3. The Association between Urinary Phthalate Metabolites and HSI Score 

The multivariate odds ratios (ORs) and 95% confidence intervals (CIs) for the HSI score according to urinary phthalate levels are shown in Table 3. The lowest level of each phthalate (first quartile) was considered as the reference value.

Urinary MEHHP and MECPP in 3rd and 4th quartiles showed higher ORs than those in the lowest quartile, in crude analysis. After adjusting for age, sex, and creatinine level (Model 1), adjusted ORs (95% CI) of MEHHP in 3rd and 4th quartiles were 1.40 (1.08–1.81) and 1.43 (1.04–1.95), compared with those in the lowest quartile.

Additionally, adjusting for smoking status, drinking status, regular exercise, marital status, education level, and socioeconomic status was performed (Model 2). The adjusted ORs of MEHHP in the 3rd and 4th quartiles were significantly higher than those in the lowest quartile (1.34 [95% CI 1.02–1.76] and 1.40 [95% CI 1.02–1.93], respectively).

When further adjusted for hypertension, DM, hyperlipidemia, and urinary bisphenol A levels (Model 3), adjusted ORs (95% CI) were 1.33 (1.00–1.77) and 1.39 (1.00–1.92) in the 3rd and 4th quartile of MEHHP, compared with those in the lowest quartile.

## 4. Discussion

This study was performed to investigate the association between urinary levels of phthalate metabolites and NAFLD in the population. The prevalence of NAFLD based on the HSI score was associated with higher levels of urinary MEHHP and MECPP in univariate analyses. After we adjusted the covariates, the 3rd and 4th quartiles of MEHHP showed significantly higher ORs compared to the lowest levels. The risk of developing NAFLD significantly increased as the quartiles of MEHHP increased in univariate and multivariate analyses.

Humans can be exposed to phthalates through ingestion (e.g., phthalate-contaminated food and water), dermal absorption (e.g., cosmetics and other personal care products), and inhalation (e.g., nail polish, hair spray, and other phthalate-containing products) [12]. Phthalates entering the body are rapidly hydrolyzed to monoesters and then metabolized in the liver and the gut [13,14,15] and excreted through the urine, with half-lives of less than 24 hours [16,17]. Phthalate monoesters represent the major human metabolites; however, the monoester of di-(2-ethylhexyl) phthalate (DEHP) is further metabolized and produced the secondary oxidized metabolites [14,16,17]. Thus, urine samples are commonly used to measure phthalate exposure.

Phthalates were classified as obesogen based on the previous animal studies on obesity and metabolic derangement [25,38,39]. Although NAFLD is closely associated with obesity [4], it also occurs in non-obese people [40,41]. That is, excess accumulation of visceral fats, which is related to IR, play a key role in NAFLD pathogenesis [41,42]. In cross-sectional studies, urinary phthalate metabolites showed an association with BMI, waist circumstances, and IR [20,21,23]. In addition, urinary MEHHP levels, which is the secondary oxidized metabolite of DEHP, showed a positive association with obesity and IR in prepubertal girls, but not with that of MEOHP and MECPP [43]. In human liver cell line, DEHP reduced insulin receptor levels and glucose oxidation, and subsequently increased IR [44]. Also, DEHP treated male rats experienced reduced insulin levels in serum and liver glycogen, and increased thyroid hormone levels in serum [22]. Because obesity and IR can contribute to the development of NAFLD [7,8,9], we hypothesized that phthalates could increase the risk of developing liver disease.

In experimental studies, exposure to DEHP, the most widely used phthalate, along with high fat or oleic acid in diets increased lipid content and inflammation in rat liver [28,33] and in a hepatocellular carcinoma cell line (HepG2 cells) [29]. The increase in hepatic lipid accumulation after phthalate exposure might be due to the up-regulation of genes related to hepatic lipid metabolism such as SREBPs and PPARs [28,29,33]. In transcriptome analysis, supporting evidence was identified for the association between exposure to a low dose of DEHP and the disruption of genes related to hepatic fatty acid metabolism in the zebrafish [30]. In addition, mono-2-ethylhexyl phthalate (MEHP), which is the primary metabolite of DEHP, increased the TG content and modulated the gene related to fatty acid metabolism in HepG2 cells [32]. In particular, perinatal exposure to DEHP induced hepatic TG content in adult male pups through up-regulation of diacylglycerol acyltransferase 1, regardless of obesity [34]. This study indicated that DEHP alone could influence re-esterification and increase small lipid droplets in the liver instead of activation of de novo lipogenesis. Although the experimental studies suggested that phthalates could accelerate lipogenesis and thereby contribute to the development of NAFLD, further study is needed to clarify the relationship between DEHP and NAFLD pathogenesis.

In addition, because thyroid hormones are important to regulate the glucose and lipid metabolism [45], the alteration of thyroid hormone levels due to phthalates may increase the risk of NAFLD. Previously, DEHP treated rats showed the decrease of thyroid hormones, sodium iodide symporter, and thyroid peroxidase levels [22,46]. It seems that DEHP can disrupt the thyroid hormone synthesis, transport, and metabolism. The negative association between phthalate metabolites levels and thyroid hormones also showed in adult men [47]. A previous meta-analysis study showed the association between hypothyroidism and NAFLD [48]. However, because the mechanism whereby phthalates induced NAFLD is associated with thyroid dysfunction is unclear, further study is needed. 

To our knowledge, this is the first study to report the association between urinary phthalate levels and prevalence of NAFLD in the general population. However, some limitations should be noted. First, we could not determine whether a noninvasive marker could truly indicate the prevalence of NAFLD. Liver biopsy is the most sensitive toll for clinical diagnosis of NAFLD. Nevertheless, this is not suitable for population-based studies. Among the various noninvasive tools for predicting NAFLD, we used the HSI score. The area under the receiver-operating curve of HSI was 0.812 (95% CI 0.801–0.824) in the Korean population [35]. Because HSI correlated with IR [49], it could estimate the phthalates induced IR, which is a primary factor of NAFLD. However, the problem of classifying the intermediate group (30 ≤ HIS ≤ 36) as a control group was an inevitable one. Further prospective studies are required to confirm the reproducibility and accuracy of our observations. Second, this study used cross-sectional survey data; thus, it was difficult to assess the causal relationship between phthalate exposure and NAFLD. Further study is warranted to investigate the influence of phthalates on NAFLD development. Third, KoNEHS represented the drinking times in the last month and the number of glass per times instead of the amount of alcohol (g/day). Heavy drinkers who consumed alcohol more than 3 times in a week and 7–9 glasses per time in men (5–6 glasses per time in women) were defined by referring to a previous study [50]. Last but not least, the inter- and intra-subject variations of urinary phthalate levels were a limitation because of the relatively short half-lives. Although KoNEHS collected single spot urine samples, a sufficient number of samples may adequately reflect the average exposure level of the population to phthalate.

## 5. Conclusions

This study showed epidemiological evidence that exposure to phthalates is associated with the occurrence of NAFLD. Therefore, a reduction in phthalate exposure might help prevent NAFLD.

## Figures and Tables

**Table 1:** General characteristics of study participants according to the hepatic steatosis index score.

Variables	Non-NAFLD (*n* = 4405)	NAFLD (*n* = 1395)	*p*-Value
Age (years)	45.79 ± 0.41	47.70 ± 0.62	0.005
Gender (%, men)	46.68 ± 0.79	50.50 ± 1.75	0.061
BMI (kg/m^2^)	22.81 ± 0.05	28.20 ± 0.10	<0.001
Drinking Status (%)			0.054
Never	29.90 ± 0.91	34.01 ± 1.69	
Former	4.97 ± 0.40	3.98 ± 0.62	
Current	65.12 ± 1.00	61.99 ± 1.79	
Smoking Status (%)			0.013
Never	65.75 ± 0.89	61.59 ± 1.78	
Former	15.23 ± 0.65	14.29 ± 1.26	
Current	19.00 ± 0.80	24.10 ± 1.69	
Physical activity (%)			0.013
No	61.43 ± 0.12	66.64 ± 1.76	
Moderate	22.06 ± 1.03	19.36 ± 1.49	
Vigorous	16.50 ± 0.82	13.99 ± 1.15	
Socioeconomic status (%)			0.040
Low	0.83 ± 0.20	1.09 ± 0.39	
Low-mid	29.17 ± 1.32	26.92 ± 2.00	
Mid-high	47.97 ± 1.22	45.01 ± 1.95	
High	22.02 ± 1.04	26.96 ± 1.91	
Education (%)			<0.001
<High school	22.99 ± 0.98	28.42 ± 1.67	
High school	38.49 ± 1.08	38.80 ± 1.83	
College and more	38.51 ± 1.23	32.76 ± 1.93	
Marital status (%)			<0.001
Single	19.67 ± 1.05	14.41 ± 1.51	
Married	72.83 ± 1.10	74.93 ± 1.75	
Divorced	7.48 ± 0.52	1.06 ± 1.21	
Comorbidity (%)			
Hypertension	12.67 ± 0.63	24.38 ± 1.54	<0.001
Diabetes mellitus	3.82 ± 0.33	15.32 ± 1.19	<0.001
Hyperlipidemia	25.01 ± 0.90	48.99 ± 1.79	<0.001

Data are shown as the weighted mean or frequency ± standard error as appropriate. NAFLD: non-alcoholic fatty liver disease; BMI: Body mass index.

**Table 2:** Urinary phthalates levels according to hepatic steatosis index score.

Concentrations (ug/L, GM ± GSE)	Total (*n* = 5800)	Non-NAFLD (*n* = 4405)	NAFLD (*n* = 1395)	*p*-Value
MEHHP	2.922 ± 0.011	2.898 ± 0.013	3.000 ± 0.023	<0.001
MEOHP	2.571 ± 0.011	2.558 ± 0.013	2.612 ± 0.023	0.031
MECPP	3.059 ± 0.010	3.039 ± 0.012	3.120 ± 0.021	<0.001
MnBP	3.211 ± 0.012	3.212 ± 0.014	3.208 ± 0.024	1.000
MBzP	1.047 ± 0.015	1.033 ± 0.017	1.091 ± 0.031	0.085

GE: geometric mean; GSE: geometric standard error; NAFLD: non-alcoholic fatty liver disease; MEHHP: mono (2-ehtyl-5-hydroxyhexyl) phthalate; MEOHP: mono (2-ethyl-5-oxohexyl) phthalate; MECPP: mono (2-ethyl-5-carboxypentyl) phthalate; MnBP: mono-*n*-butyl phthalate; MBzP: mono-benzyl phthalate.

**Table 3:** The association between hepatic steatosis index score and urinary phthalates levels (ug/L).

Phthalate Metabolites	Crude		Model 1		Model 2		Model 3	
	OR (95% CI)	*p*-Value	OR (95% CI)	*p*-Value	OR (95% CI)	*p*-Value	OR (95% CI)	*p*-Value
MEHHP								
Quartile 1	1	<0.001 *	1	0.008 *	1	0.015 *	1	0.019 *
Quartile 2	1.09 (0.85–1.40)	0.482	1.09 (0.83–1.44)	0.514	1.07 (0.81–1.42)	0.594	1.05 (0.80–1.38)	0.702
Quartile 3	1.39 (1.14–1.71)	0.001	1.40 (1.08–1.81)	0.010	1.34 (1.02–1.76)	0.030	1.33 (1.00–1.78)	0.044
Quartile 4	1.41 (1.12–1.77)	0.003	1.43 (1.04–1.95)	0.024	1.40 (1.02–1.93)	0.035	1.39 (1.00–1.92)	0.044
MEOHP								
Quartile 1	1	0.069 *	1	0.657 *	1	0.784 *	1	0.835 *
Quartile 2	1.10 (0.86–1.40)	0.445	1.04 (0.80–1.35)	0.751	1.03 (0.79–1.34)	0.784	1.01 (0.77–1.31)	0.929
Quartile 3	1.12 (0.98–1.51)	0.067	1.10 (0.83–1.46)	0.480	1.07 (0.80–1.44)	0.618	1.05 (0.77–1.43)	0.732
Quartile 4	1.19 (0.96–1.49)	0.109	1.05 (0.78–1.42)	0.723	1.03 (0.76–1.40)	0.827	1.02 (0.74–1.39)	0.888
MECPP								
Quartile 1	1	0.020 *	1	0.214 *	1	0.267 *	1	0.245 *
Quartile 2	1.07 (0.85–1.36)	0.531	1.04 (0.80–1.35)	0.729	1.03 (0.80–1.34)	0.773	1.01 (0.77–1.31)	0.935
Quartile 3	1.28 (1.04–1.59)	0.020	1.20 (0.91–1.58)	0.179	1.17 (0.88–1.55)	0.270	1.14 (0.85–1.54)	0.365
Quartile 4	1.26 (1.00–1.60)	0.047	1.18 (0.86–1.62)	0.287	1.17 (0.84–1.61)	0.337	1.18 (0.85–1.65)	0.311
MBzP								
Quartile 1	1	0.533 *	1	0.664 *	1	0.710 *	1	0.792 *
Quartile 2	1.08 (0.86–1.37)	0.467	1.02 (0.80–1.30)	0.853	1.07 (0.84–1.36)	0.573	1.05 (0.82–1.34)	0.673
Quartile 3	1.14 (0.92–1.42)	0.220	1.03 (0.80–1.32)	0.711	1.08 (0.84–1.38)	0.529	1.06 (0.82–1.35)	0.641
Quartile 4	1.06 (0.84–1.34)	0.612	0.93 (0.70–1.23)	0.626	0.94 (0.71–1.24)	0.678	0.95 (0.72–1.26)	0.769
MnBP								
Quartile 1	1	0.860 *	1	0.191 *	1	0.304 *	1	0.420 *
Quartile 2	1.17 (0.95–1.44)	0.139	1.05 (0.83–1.34)	0.630	1.08 (0.85–1.38)	0.492	1.07 (0.83–1.37)	0.571
Quartile 3	1.08 (0.86–1.34)	0.481	0.90 (0.68–1.18)	0.449	0.93 (0.71–1.22)	0.632	0.94 (0.72–1.23)	0.685
Quartile 4	1.05 (0.82–1.33)	0.686	0.83 (0.60–1.15)	0.279	0.87 (0.63–1.21)	0.424	0.90 (0.64–1.25)	0.540

OR: odds ratio; CI: confidence interval; MEHHP: mono (2-ehtyl-5-hydroxyhexyl) phthalate; MEOHP: mono (2-ethyl-5-oxohexyl) phthalate; MECPP: mono (2-ethyl-5-carboxypentyl) phthalate; MnBP: mono-*n*-butyl phthalate; MBzP: mono-benzyl phthalate. *: *p* values were shown the test of trend of odds. Crude: hepatic steatosis index, each type of phthalate metabolites. Model 1: Crude + age, sex, creatinine. Model 2: Model 1 + smoking, drinking, exercise, marital status, education, socioeconomic status. Model 3: Model 2 + hypertension, diabetes mellitus, hyperlipidemia.

## Data Availability

This study used data from the Second Korean National Environmental Health Survey (KoNEHS) which was conducted by Ministry of Environment, National Institute of Environmental Research. The data presented in this study are available on request from the corresponding author. The data are not publicly available due to protect personal information.
